# A physics informed neural network approach to quantify antigen presentation activities at single cell level using omics data

**DOI:** 10.21203/rs.3.rs-5629379/v1

**Published:** 2025-01-17

**Authors:** Chi Zhang, Jia Wang, Pengtao Dang, Yuhui Wei, Xiao Wang, Julie Brothwell, Yifan Sun, Haiqi Zhu, Kaman So, Jing Liu, Yijie Wang, Xiongbin Lu, Stanley Spinola, Xinna Zhang, Sha Cao

**Affiliations:** Indiana University School of Medicine; Indiana University; Oregon Health & Science University; Oregon Health & Science University; Indiana University; Indiana University School of Medicine; Indiana University School of Medicine; Indiana University; Indiana University School of Medicine; Purdue University; Computer Science Department, Indiana University; Zhejiang University; Indiana University School of Medicine; Indiana University School of Medicine; Oregon Health & Science University

## Abstract

Antigen processing and presentation via major histocompatibility complex (MHC) molecules are central to immune surveillance. Yet, quantifying the dynamic activity of MHC class I and II antigen presentation remains a critical challenge, particularly in diseases like cancer, infection and autoimmunity where these pathways are often disrupted. Current methods fall short in providing precise, sample-specific insights into antigen presentation, limiting our understanding of immune evasion and therapeutic responses. Here, we present PSAA (PINN-empowered Systems Biology Analysis of Antigen Presentation Activity), which is designed to estimate sample-wise MHC class I and class II antigen presentation activity using bulk, single-cell, and spatially resolved transcriptomics or proteomics data. By reconstructing MHC pathways and employing pathway flux estimation, PSAA offers a detailed, stepwise quantification of MHC pathway activity, enabling predictions of gene-specific impacts and their downstream effects on immune interactions. Benchmarked across diverse omics datasets and experimental validations, PSAA demonstrates a robust prediction accuracy and utility across various disease contexts. In conclusion, PSAA and its downstream functions provide a comprehensive framework for analyzing the dynamics of MHC antigen presentation using omics data. By linking antigen presentation to immune cell activity and clinical outcomes, PSAA both elucidates key mechanisms driving disease progression and uncovers potential therapeutic targets.

## Introduction

Antigen processing and presentation are fundamental mechanisms within the immune system, wherein intact protein antigens undergo degradation to produce peptide fragments that are loaded onto major histocompatibility complex (MHC) molecules and presented on the cell surface of antigen-presenting cells (APCs), further facilitating recognition by T cells. Humans have two different classes of antigen presentation molecules: MHC class I (MHC-I) molecules mainly bind to endogenous antigens. MHC-I is expressed by all nucleated cells throughout the body and recognized by CD8 + cytotoxic T lymphocytes (CTLs). MHC class II (MHC-II) is primarily expressed by professional APCs, mainly bind to exogenous antigens, and are recognized by CD4 + cells.

Functional variations in MHC-I and MHC-II have been reported in different disease conditions and cell types. Immune evasion is a hallmark feature of cancer that enables the cancer cells to escape immune recognition and destruction and is often achieved through the reduction or loss of antigen presentation ^1–3^. Immunotherapy, such as checkpoint blockade treatments, boosts T cell-mediated immune responses ^4–6^. Immunotherapy relies on the recognition of tumor cells by CTLs, which recognize tumor cells based on tumor associated antigens (TAA) presented on surface of cancer cells via MHC-I ^7–9^. Impairing this process will ultimately diminish or eliminate CD8 + T cell-mediated tumor cytotoxicity and cause treatment resistance. The MHC complex also plays a significant role in various other diseases, primarily those related to immune system dysfunction^10, 11^. In autoimmune diseases such as rheumatoid arthritis, multiple sclerosis, and lupus, MHC-II antigen presentation dysregulation leads to the presentation of self-antigens to T cells, triggering an inappropriate immune response against the body’s own tissues^12^. Some intracellular pathogens can evade immune responses by interfering with pathways involved in MHC-II antigen presentation^13^. Diseases characterized by chronic inflammation, such as inflammatory bowel disease (IBD), psoriasis, or even cancer, also involve dysregulated MHC-II antigen presentation, contributing to sustained immune activation and tissue damage^10, 14, 15^.

While substantial efforts have been focused on studying the source and quality of antigens, the activity level and impairment of the MHC-I and MHC-II pathways have been understudied in disease-specific contexts^16^. To the best of our knowledge, there is a lack of a method that can accurately characterize the dyanamics of the MHC-I and MHC-II antigen presentations and their regulators using omics data. To fill this gap, we developed a computational method named Physics Informed Neural Network (PINN)-empowered Systems Biology Analysis of Antigen Presentation Activity (PSAA) to estimate the flux of MHC class I and II antigen presentation pathways at the individual sample or cell resolution using transcriptomics or proteomics data.

PINN is a novel learning framework that combines data-driven machine learning models with governing physical laws, encoded as differential equations, to enhance predictions in complex biological systems ^17, 18^. By incorporating known biological constraints, PINNs allow for more accurate and interpretable modeling of biological processes than conventional data driven approaches using omics data. However, directly applying PINNs to estimate the dynamics of antigen presentation presents challenges due to the static nature of transcriptomics and proteomics data, which lack temporal dynamics and may not capture real-time regulatory fluctuations within MHC pathways. This limitation necessitates careful modeling assumptions to infer pathway activity from such static snapshots.

PSAA was empowered by a new PINN framework. One neural network was trained for each reaction step in the MHC-I or -II to approximate its sample-wise flux rate by leveraging the goodness of fit of their underlying systems biology model with observed omics data. Specifically, the MHC-I and MHC-II pathways consist of large molecular processing reactions that can be treated as enzyme-catalyzed reactions, in which proteins, peptides, antigens, and MHC complexes across different steps and subcellular localizations act as intermediate substrates. In this context, the antigen source serves as the input, while the MHC complexes presented on the cell surface, or further T cell recognition levels, can be considered as the outcome of the pathway. Under this framework, the system operates under steady-state equilibrium, which PSAA leverages to estimate sample-wise MHC-I and MHC-II antigen presentation levels, as well as the impact of each gene involved in the antigen presentation approach for each individual sample.

To develop PSAA, we first curated and reconstructed the MHC-I and MHC-II pathways by collecting genes involved in each reaction step from multiple pathway databases and literature. Based on the steady-state equilibrium hypothesis, we developed a new flux estimation and optimization method to assess the activity level of each step of the MHC-I and MHC-II pathways in each sample using omics data. PSAA, along with its downstream functions, offers several capabilities: (1) predicting the activity level of the entire process and individual biological steps of the MHC-I and MHC-II pathways in each sample using diverse types of transcriptomics or proteomics data, (2) assessing the impact of each individual gene or step on MHC-I and MHC-II antigen presentation, (3) evaluating the association of MHC-I and MHC-II antigen presentation with T cell activity and other biological or clinical features, and (4) inferring potential regulators or varied network structures of the MHC-I and MHC-II pathways in specific disease or tissue contexts. We rigorously benchmarked the prediction accuracy, robustness, and utility of PSAA using a broad spectrum of public omics data including bulk, single-cell, and spatially resolved transcriptomics or proteomics data of cancer, inflammatory, and neurodegenerative conditions. We also generated matched scRNA-seq and SRT data of bacterial infected human skins vs wound to further demonstrate the application of PSAA in analyzing varied antigen presentation mechanisms under different inflammatory conditions. To validate the prediction of PSAA, we conducted CRISPR knock-down experiments of key genes predicted by PSAA to amplify MHC-I antigen presentations in cancer.

## Results

### The overall framework of PSAA.

**Figure 1:**
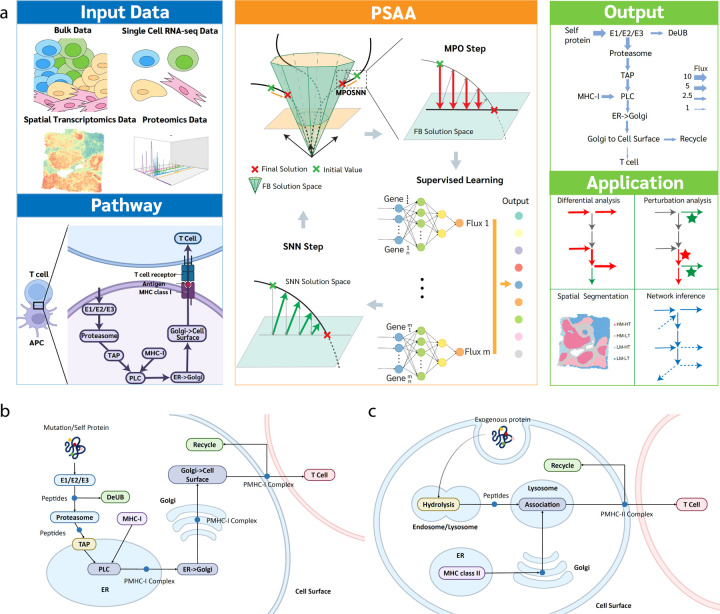
Overview of the PSAA framework. (a) The analysis pipeline of PSAA. The initial setup of PSAA includes input data and selected antigen presentation pathway. As validated in this study, the input data can be bulk transcriptomics data, scRNA-seq data, spatial transcriptomics data, or proteomics data and the selected pathway can be MHC-I or MHC-II pathways. The core algorithm of PSAA consists of four steps: (1) computing initial flux; (2) projecting the current flux into the flux balance solution space (MPO step); (3) training a neural network to predict the MPO-balanced flux using the input data (SL step); and (4) iteratively conducting steps (2) and (3) until convergence. The output of PSAA is sample-wise activity level of each step in the MHC-I and MHC-II pathways. Downstream applications of PSAA include analyses of differential flux, in-silico perturbation, spatial segmentation, cell-cell interactions, and pathway structure. (b) Reconstructed MHC-I antigen presentation pathway. Rounded boxes represent reactions, and blue dots represent intermediate products. (c) Reconstructed MHC-II antigen presentation pathway.

PSAA is a new PINN-based model to quantify sample-wise MHC class I and II antigen presentation activity using transcriptomics or proteomics data. As illustrated in Fig. 1a, PSAA takes bulk, single-cell, or spatially resolved data as the input to approximate the reaction rate of each step in the MHC-I or MHC-II antigen presentation pathways in each sample. PSAA utilizes a new optimization approach, namely message passing and supervised learning (MPSL), to compute the non-linear dependency between gene expression and reaction rate by leveraging (i) the flux balance of the reaction flows and (ii) the goodness of fit to the data. The output of PSAA provides the sample-wise reaction rates for each step in the selected MHC-I or MHC-II pathway. These rates could be directly used to explore potential associations with other molecular or clinical features, to study the functional role or clinical implications of antigen presentation in the disease context. PSAA also enables downstream analysis, including: (1) evaluating the differences in MHC-I or MHC-II antigen presentation across different disease contexts and their correlations with other features, (2) conducting in-silico perturbation analysis to identify genes with the greatest context-specific impact on the antigen presentation pathway to highlight potential drug targets, (3) performing antigen presentation and recognition-informed spatial segmentation for spatially resolved transcriptomics (SRT) data to analyze interactions between APCs and T cells, and (4) inferring with network structure and potential regulators of antigen presentation and T cell recognition.

### Reconstruction of MHC-I and MHC-II antigen presentation pathways.

To accurately estimate the activity level of antigen processing and presentation, we first reconstructed the MHC-I and MHC-II pathways and related branches, ensuring they recapitulate all molecules or their complexes serving as sources, products, and facilitators of all the involved reactions (Fig. 1b, c). Although databases such as the Kyoto Encyclopedia of Genes and Genomes^19^ (KEGG), REACTOME^20^, and GO^21^ provide sets of genes related to antigen presentation, to the best of our knowledge, there is a lack of fully annotated MHC-I and MHC-II pathways that comprehensively cover the individual antigen processing, presentation, and salvage steps for systems biology analysis. Thus, we first collected genes that are involved in the MHC-I and MHC-II pathways and related biological processes via integrating the pathways annotated in these databases and further curated the integrated pathway by an extensive literature search ^7, 22–24^.

Our reconstructed MHC-I pathway consists of eight reaction steps, namely ubiquitination, proteasomal degradation, the action of the transporter associated with antigen processing (TAP), chaperone-assisted assembly of the MHC class I complex, transit from the endoplasmic reticulum (ER) to the Golgi apparatus, and subsequent exocytosis from the Golgi to the cell membrane. Additionally, auxiliary pathways such as de-ubiquitination, verification of MHC class I complex integrity, and recycling (salvage) of cell surface MHC-I complex through endocytosis were also added as branches in this pathway. The final MHC-I antigen presentation pathway includes 284 genes in eight reaction modules.

The final MHC-II antigen presentation pathway consists of seven reaction modules, including uptake of extracellular proteins into cells, processing of internalized proteins in endosomal/lysosomal compartments, biosynthesis of MHC-II complex in the ER, transport of MHC-II from the ER to the Golgi, transport of MHC-II from the Golgi to Endosomes, association of MHC-II with antigen, and expression of peptide-MHC-II complexes on the cell surface. The reconstructed MHC-II pathway includes 124 genes in seven reaction modules.

Notably, the reconstructed MHC-I and MHC-II pathways were optimized for a systems biology-model based estimation of pathway activity level, which have the following features: (1) the pathways cover the main antigen processing and presentation steps as well as side branches such as de-ubiquitination and recycling of cell surface MHC complexes; (2) the reaction steps within the reconstructed networks were optimized to reduce duplicated genes in adjacent steps; and (3) the reconstructed pathways were represented as directed factor graphs where each intermediate state of antigen or MHC complexes is a factor and each reaction step is a variable. The PINN model was further developed over the directed factor graphs to estimate the activity level of each reaction step using expression changes of the genes or proteins involved in the steps. Detailed pathway reconstructions are provided in Supplementary Methods, and the reconstructed MHC-I and MHC-II pathways are given in Supplemental Tables S1 and S2.

### Systems biology considerations, mathematical model, and solution of PSAA.

PSAA is built upon the PINN framework that approximates the sample-wise activity level of MHC-I and MHC-II pathways using transcriptomics or proteomics data. PSAA first hypothesizes that the rate of each reaction of the MHC-I and MHC-II pathway could be inferred from the transcriptomics or proteomics changes of the involved genes via unknown non-linear functions. To train these functions, we first analyzed the systems properties of the MHC pathways. All reactions in the MHC-I and MHC-II pathways are large molecule processing reactions, in which proteins, peptides, MHC class I or II complexes, and their binding forms are substrates or products of each reaction step. The whole pathway satisfies the law of conservation of mass under steady or quasi-steady state, known as the flux balance condition, which has been widely utilized in metabolic flux analysis^25–27^. Thus, PSAA further assumes that the predicted activities of each reaction in the MHC-I and MHC-II pathway should satisfy a quasi-flux balance condition.

PSAA approximates reaction rates using non-time course omics data, which differs from traditional PINN models. To enable a robust and explainable prediction, we developed a new learning paradigm—**constrained learning**—and a new optimization algorithm, as detailed below and in Methods. Given an omics data set D and a pathway of m reactions, denote the flux rate of the *ith* reaction $\left(i=1,\ldots m\right)$ in the MHC-I or –II pathways in the *jth* sample in D as $F_{i,j}$. PSAA identifies functions ${𝓕}_{i}$ to estimate $F_{i,j}$ by $F_{i,j}={𝓕}_{i}\left(D_{,j},\Theta^{i}\right)$ and minimizes a loss function $L=L_{FB}\left({\hat{F}}_{,j}\right)+L_{p}\left({𝓕}_{i},\Theta_{i}|i=1,\ldots ,m\right)$, where ${\hat{F}}_{,j}≜\left\{{\hat{F}}_{i,j}|i=1,\ldots ,m\right\}$ denotes the predicted reaction rates in sample j, $\Theta_{i}$ denotes parameters of ${𝓕}_{i}$, $L_{FB}$ is a quadratic loss term that regularizes the imbalance of predicted reaction rate, and $L_{p}$ an aggregated computational loss term to avoid trivial solutions and to conduct variable selection.

Recognizing that the PSAA framework is neither supervised nor unsupervised learning, we developed a new optimization approach called MPSL (Message Passing enhanced Supervised Learning) to enable a robust and efficient solution of ${𝓕}_{i}$ and $\Theta^{i}$. As illustrated in Fig. 1a, a two-step optimization approach is introduced to iteratively (1) Message Passing Optimization (MPO) step: Computing ${\hat{F}}_{,j}^{t+1}$ that minimizes $L_{FB}\left({\hat{F}}_{i,j}^{t+1}\right)$ by searching through $L_{FB}\left({\hat{F}}_{,j}^{t+1}\right)$ within a certain distance to ${𝓕}_{j}=\left(D_{,j},\Theta_{i}^{t}\right)$ and (2) Supervised Learning (SL) step: Updating $\Theta_{i}^{t+1}$ by conducting a supervised training of ${𝓕}_{i}=\left(D_{,j},\Theta_{i}^{t+1}\right)$ to estimate $L_{FB}\left({\hat{F}}_{,j}^{t+1}\right)$, where t and t+1 denote successive rounds of iterations. A key challenge of PINN is to balance fit to both physical models and data when using neural networks to approximate the non-linear dependency. The MPSL algorithm addresses this challenge by splitting the fitting process into two iterative steps: fitting the physical model and fitting the data. This makes the data-fitting step a classic supervised learning problem that can be implemented with additional regularization terms for variable selection or better fitting robustness. The MPSL algorithm was validated on an extensive set of simulated data (see details in Supplementary Methods). The detailed mathematical formulation of the MPSL optimization algorithm is given in Methods.

#### Downstream functionalities of PSAA

We also developed a series of downstream functionalities of PSAA. The outputs of PSAA, i.e., sample-wise activity levels for each step in the MHC-I and MHC-II pathways can be directly compared across different contexts. Because ${𝓕}_{i}=\left(D_{,j},\Theta^{i}\right)$ directly utilizes neural networks to approximate sample-wise reaction rate, its partial derivative $\frac{\partial {𝓕}_{i}}{\partial D}$ could be computed to evaluate the impact of each gene in each sample. In-silico perturbation analysis can be conducted to identify genes that, when perturbed, may increase or decrease antigen presentation levels. In spatial transcriptomics data, the predicted MHC-I and MHC-II activity level can be directly utilized to characterize the interaction between APCs and T cells, enabling an APC-T cell interaction-based spatial dissection. Additionally, the coherence between the MPO and SL steps can be used to evaluate the goodness of fitting of the data to the systems biology assumption and pathway structure. This coherence can be further used to infer the pathway structure variation.

We further validated PSAA and its downstream functions using public data and experimental approaches. The MPSL optimization was validated using a set of synthetic data-based experiments. We also demonstrated the application of PSAA and its functions for different antigen-presentation disease scenarios including cancer, bacterial infection, and neurodegeneration.

### PSAA accurately quantifies antigen presentation activity using transcriptomics or proteomics data.

**Figure 2:**
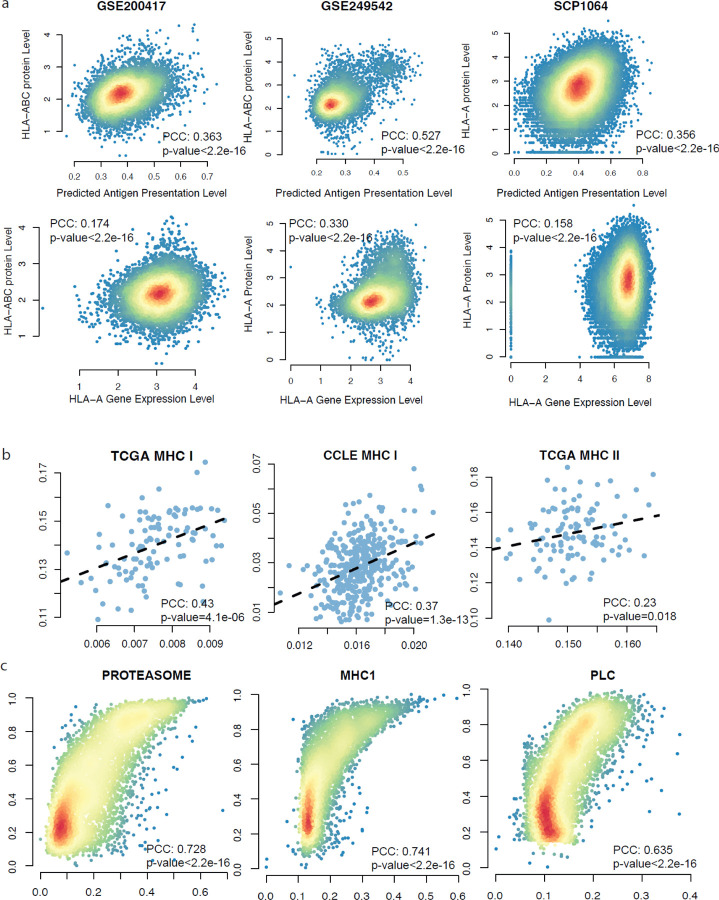
Validation of the prediction accuracy of PSAA. (a) 1^st^ Row: Consistency of the PSAA predicted MHC-I antigen presentation level (x-axis) vs experimentally measured cell surface MHC-I complex level (y-axis) in three CITE-seq data and TEA-seq data. 2^nd^ Row: Consistency of the expression level of MHC-I genes (x-axis) vs experimentally measured cell surface MHC-I complex level (y-axis). (b) Consistency of the PSAA predicted MHC-I and II level using RNA-seq data (x-axis) and proteomics data (y-axis) in TCGA and CCLE. (c) Consistency of the predicted activity level of different steps in MHC-I pathway predicted using TCGA data by including (x-axis) and excluding (y-axis) the T cell recognition step in the input pathway.

To benchmark PSAA, we first tested the Pearson correlation coeficients (PCC) between the predicted antigen presentation level and the cell surface protein of MHC-I molecules in three independent CITE-seq and TEA-seq data sets (CITE-seq of B-cell malignancies: GSE249542, CITE-seq of melanoma: SCP1064^28^, and TEA-seq of T cells: GSE200417^29^). Both CITE-seq and TEA-seq offer simultaneous measurement of gene expression and protein levels. We found that PSAA-predicted antigen presentation levels were significantly associated with measured cell surface MHC I molecules in all three datasets: GSE200417, PCC = 0.363 (p-value < 2.2e-16); GSE249542, PCC = 0.527 (p-value < 2.2e-16), and SCP0164, PCC = 0.356 (p-value < 2.2e-16), as shown in Fig. 2a (first row). Noted, these correlations were much higher than those that use averaged HLA class I or II gene expressions to predict antigen presentation activity Fig. 2a (second row). Our results demonstrate that PSAA could accurately predict cell surface MHC complex presentation levels.

To evaluate the robustness of PSAA, we applied PSAA to the CCLE and TCGA matched bulk RNA-seq and proteomics data sets, respectively, to test the consistency of the predictions made from transcriptomics and proteomics data. We identified a significant consistency between the predictions made from the two data sources for both MHC-I (PCC = 0.43, p-value = 4.1e-06) and MHC-II (PCC = 0.23, p-value = 0.018) antigen presentation pathways in the TCGA data (Fig. 2b). In CCLE data, we identified a significant consistency of the MHC-I (PCC = 0.37, p-value = 1.3e-13) pathway (Fig. 2b) and MHC-II pathway (PCC = 0.12, p-value = 0.02) (Supplementary Fig. 1a). Noted, a weaker correlation of the predicted MHC-II activity in the CCLE data is expected, since the CCLE data set is derived from pure cancer cells that normally do not present MHC-II molecules. Overall, our observation demonstrated that PSAA could robustly predict MHC-I and MHC-II antigen presentation levels using either transcriptomics or proteomics data.

To assess PSAA’s robustness, we conducted an ablation study by removing the “T cell recognition” module at the end of the antigen presentation pathway and compared with results obtained from the original pathway (Fig. 2c and Supplementary Fig. 1b). While including the “T cell recognition” module intuitively enhances the completeness of the pathway, and could yield more accurate predictions, we observed significant consistency between the predicted flux of each step when using a network with and without the T cell module (PCC = 0.635 in PLC, PCC = 0.728 in proteasome, and PCC = 0.741 in MHC-I). This indicates that PSAA can accurately quantify antigen presentation levels even without considering the “T cell recognition” module. Notably, this analysis demonstrated that PSAA could robustly estimate antigen presentation without considering neighboring T cell infiltration levels, making it suitable for applications in single-cell or spatial transcriptomics data, where individual samples (single cell or spatial spot) may not contain information about neighboring T cells.

To further analyze PSAA’s robustness to data sparsity, missing data, and overfitting, we conducted a systematic evaluation on CITE-seq data of peripheral blood mononuclear cells (PBMCs) (GSE249542). We first analyzed the impact of potential dropout events in scRNA-seq or SRT data by simulating different levels of dropout in the input scRNA-seq data. Our results suggested that PCC was highly robust to dropouts (Supplementary Fig. 1c). Similarly, we evaluated the robustness of PCC to missing genes (Supplementary Fig. 1c). We also conducted statistical analysis of the necessary input sample size for PSAA, evaluated overfitting by permutation test, and conducted robustness tests of PSAA (see details in Supplementary Methods). Using simulated data, MPSL always projected a high-dimensional flux vector to the closest point in the solution space of flux balance (see Supplementary Methods).

### PSAA accurately estimates MHC II antigen presentation activity and APC-T cell interactions in a bacterial infection model of human volunteers.

**Figure 3:**
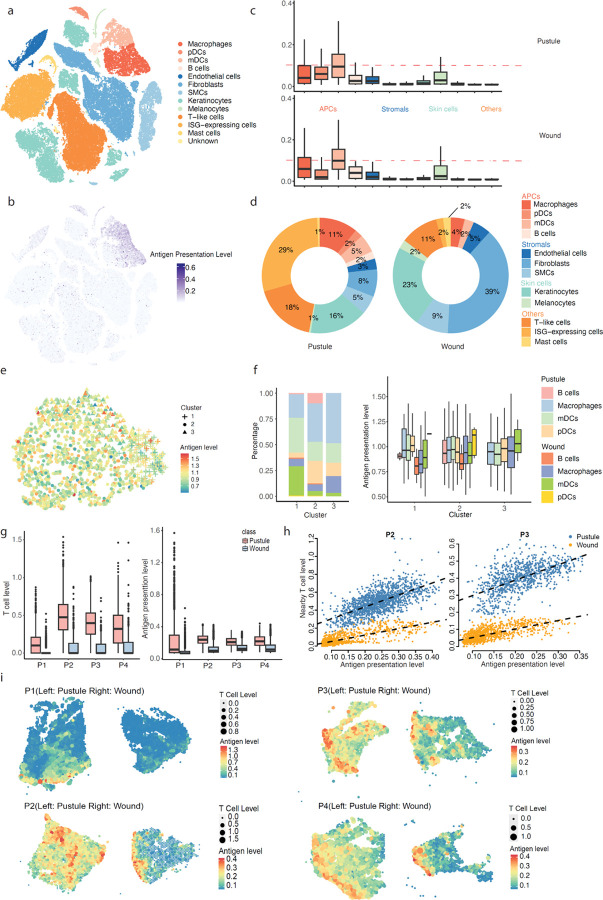
Application of PSAA and downstream functions on matched single cell and spatial transcriptomics data obtained from *Haemophilus ducreyi* infected skin and uninfected (wounded) skin. (a) tSNE of the distribution of scRNA-seq data and cell types. (b) Distribution of PSAA predicted MHC-II antigen presentation level in each single cell over the tSNE of the scRNA-seq data. (c) Distribution of PSAA predicted cell surface MHC-II antigen presentation level (y-axis) in each cell type (x-axis) on scRNA-seq data. Cell types are colored as (a) and (d). (d) Proportion of each cell type in the pustules and wounds. (e) tSNE plot of APCs and the three APC cell clusters derived using the first-order partial derivative of each gene in the MHC-II pathway. (f) Proportion of APC cell types in the three clusters (left) and PSAA predicted MHC-II whole pathway activity level of each cell type in the three clusters (right). (g) T cell level (left) and PSAA predicted MHC-II whole pathway activity level (right) in the spatial spots of the SRT data. (h) Varied dependency between T cell level and MHC-II antigen presentation level in the SRT data of pustules (blue) vs wounded skin (orange) in patient sample 1 and 2. (i) Distribution of T cells and predicted MHC-II antigen presentation level on the spatial slides.

To further validate PSAA and demonstrate its application in studying the variation of MHC-II pathway in antigen-presenting cells (APCs), we generated a matched scRNA-seq and SRT data sets derived from skin biopsies of four human volunteers. The volunteers provided paired biopsies of skin sites that were wounded (uninfected controls) or inoculated via puncture wounds with *Haemophilus ducreyi* until a pustule developed at that site 6–8 days later (see details in Supplementary Methods). In the scRNA-seq data, there were four types of APCs (macrophages, pDCs, mPCs, and B cells), two types of skin cells (keratinocytes and melanocytes), three types of stromal cells (endothelial cells, fibroblast cells, and smooth muscle cells), three other immune cell types (ISG-expressing cells, T cells and mast cells), and one unknown cell group (Fig. 3a). PSAA was applied to approximate cell surface MHC-II antigen presentation activity in each cell except for those in the unknown cluster (Fig. 3b). PSAA identified that the APCs have the highest level of MHC-II antigen presentation activity, followed by endothelial cells and melanocytes, which have an intermediate level of MHC-II antigen presentation activity, and the other immune cell types that have relatively low antigen presentation activity (Fig. 3c). We also found that the pDCs in pustules have a higher level of MHC-II presentation compared to wounds (p = 4e-12) while the other cell types do not have a significant difference (Fig. 3c). In addition, we see a consistent increase of cell proportions of APCs in pustules vs wounds (Fig. 3d). The predicted lack of antigen presentation by pDCs and decreased population of macrophages in pustules may reflect the failure of the immune response to clear infection and inability to detect *H. ducreyi*-specific antibodies after infection ^30^. The increased MHC-II antigen presentation in pustules compared to wounds likely reflects the presence of exogenous antigen and consequent influx of immune cells to the site of infection in pustules; the immune influx is largely absent in wounds.

We also applied the in-silico perturbation analysis to study which genes contribute most to MHC-II antigen presentation pathways in APCs. The first-order partial derivative of each gene in the PSAA model was computed for each sample, which evaluates the impact of the gene to the pathway activity level if its expression level is disrupted. The partial derivatives of each gene in each reaction step were analyzed to identify if the genes contribute to MHC-II antigen presentation differently (Fig. 3e, see details in Methods). Cell clustering analysis using the partial derivatives identified three APC subgroups that potentially have different MHC-II antigen presentation mechanisms: namely, *APC-G1*, represented by mDCs in both infected and wounded sites; *APC-G2*, consisting of APC cells specifically in infected sites; and *APC-G3*, represented by macrophages in both infected and wounded sites (Fig. 3f). Perturbation analysis suggested that the whole activity level of the MHC-II pathway in *APC-G1* mostly determined by *HLA-DR*, *HLA-DP*, and *HLA-DQ*. MHC II in APC-G2 is mostly determined by *CD74*, *HLA-DM*, and *HLA-DR*, and the C1 peptidases *CTSZ* and *CTSC*, and *RAB14*, and MHC-II in APC-G3 specifically depends on the C1 peptidases *CTSZ*, *CTSB*, *CTSS*, *CTSD*, *CTSH*, and *CTSL* (Supplementary Table S3). Only the cells in the APC-G1 group had a significantly increased antigen presentation level in pustules versus wounds (Fig. 3f). Based on differential gene expression and perturbation analysis, the varied expression in the MHC II genes *HLA-DR*, *HLA-DP*, and *HLA-DQ* drove the differences between cells in the infected and wounded sites.

By PSAA to the SRT data, we observed again that pustules have significantly increased MHC-II antigen presentation activity and CD4 + helper T cells than wounded tissues (Fig. 3g). Spatial dependent regression analysis revealed that the CD4 + helper T cells level significantly depends on MHC-II activity in both conditions (Fig. 3h and Supplementary Fig. 2), and donors showed a higher level of activation dependency (larger slope) between MHC-II antigen presentation and CD4 + T cells in pustules than in wounded skin ^31^. The strong linear dependency suggests a strong colocalization of APCs with CD4 + T cells in both conditions while the varied dependencies suggest a stronger CD4 + T cell activation and increased adaptive immune response in pustules than in wounded skin (Fig. 3h). In pustules, MHC-II antigen presentation was more diffused throughout the skin; in wounds, high MHC-II activity spots were enriched in the surface regions (epidermis) (Fig. 3i). A similar pattern was observed for CD4 + helper T cells (Fig. 3i).

Our analysis demonstrates the potential utility of PSAA in the integrative analysis of scRNA-seq and SRT data. This approach facilitates the assessment of MHC-II activity and further subtyping of APCs, as well as the interpretation of biological functional variations such as antigen presentation mechanisms, spatial distribution of MHC-II antigen presentation, and spatial dependent MHC-II – T cell interactions.

### PSAA captured MHC-I antigen presentation level in cancer tumor microenvironment and identified potential targets to improve CD8 + T cell’s recognition and cytotoxicity.

**Figure 4:**
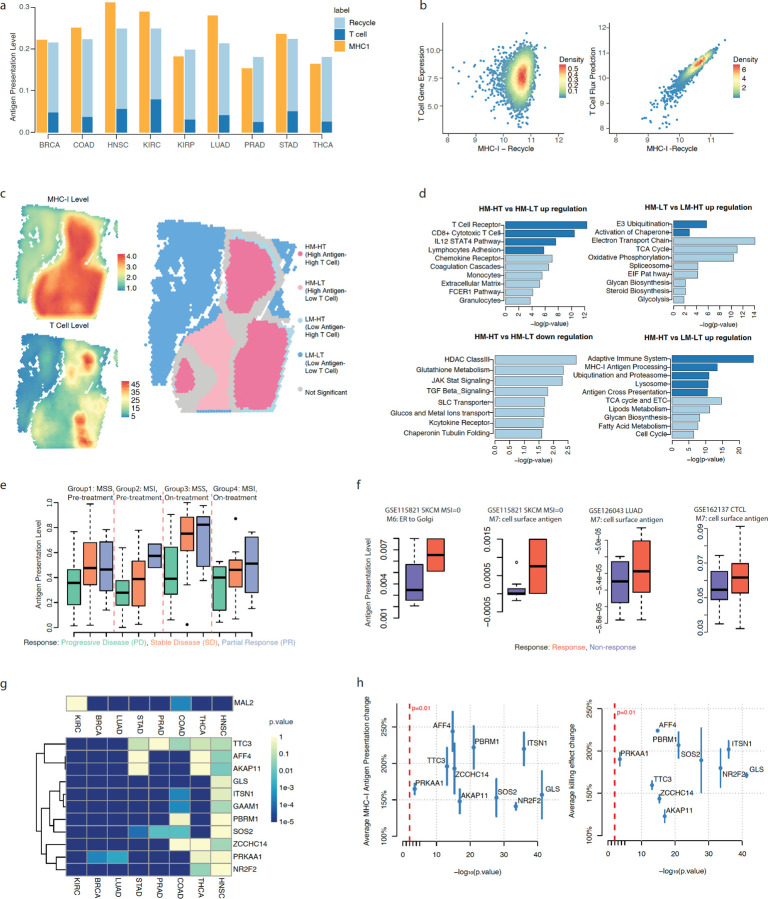
Application of PSAA and downstream functions on TME data. (a) PSAA predicted MHC-I antigen presentation on the cell surface vs CTL level + recycling of MHC-I across nine cancer types. (b) Mean CTL cell gene expression level vs MHC-I level - recycling of MHC-I (left) and CTL cell flux prediction level vs MHC-I level - recycling of MHC-I (right). (c) Spatial dissection conducted by using PSAA predicted MHC-I level and T cell level. (d) Pathway enrichment of the differentially expressed genes between different spatial regions. Pathways that directly relate to our collected MHC-I antigen presentation pathway are marked as dark blue. (e) MHC-I antigen presentation level in patients of different responses to PD-1 inhibitors. Progressive Disease (PD), Stable Disease (SD) and Partial Response (PR) groups are colored green, orange, and blue, respectively. Group 1: MSS and pre-treatment patients; Group 2: MSI and pre-treatment patients; Group 3: MSS and on-treatment patients; and Group 4: MSI and on-treatment patients. (f) PSA predicted antigen presentation levels in response vs non-response patients in four independent data sets. (g) p-values of *MAL2* and the genes that are predicted to have the most negative impacts on the MHC-I antigen presentation in 8 TCGA cancer types. (h) Increase of antigen presentation level (y-axis, left) and T cell killing effect (y-axis, right) by CRISPR knocking-down the genes predicted to have the most negative impact on MHC-I antigen presentation. Here x-axis is the -log(p-value) of the impact predicted by using TCGA breast cancer type.

Immunotherapy has shown remarkable eficacy in treating multiple cancer types^4–6^. However, mechanisms underlying non-responsiveness, particularly in solid tumors, remain poorly understood^32–36^. In the context of CD8 + cytotoxic T lymphocytes (CTL) – mediated immune responses, recognition of tumor associated antigen occurs through its presentation via MHC-I molecule on tumor cells and their interaction with T cell receptor (TCR) on the CD8 + T cells ^7–9^. Impairing this event will ultimately reduce or prevent CD8 + T cell mediated tumor cytotoxicity. However, reduction or loss of antigen presentation is a frequent mechanism used by tumor cells to escape immune recognition and destruction ^1–3^. Little is known regarding how and what variations of gene expression or key biological steps in the MHC-I pathway affect the level of antigen presentation on the surface of cancer cells and their downstream recognition by T cells. Therefore, we applied PSAA to predict how MHC-I antigen presentation affects the immune responses in cancer. We first validated the application of PSAA on TCGA RNA-seq data from nine cancer types. The T cell level could be well explained by the activity level of MHC-I antigen presentation and its recycling (Fig. 4a). However, the T cell level alone poorly recapitulates MHC-I activity (Fig. 4b). Further analysis suggested that T cell activation via MHC-I is increased in the cancer TME compared to the adjacent normal tissues although both cancer and normal tissues have a similar amount of the MHC-I presentation (Supplementary Fig. 3a). Our analysis revealed that the majority of the presented MHC-I tend to be recycled in the TME. This observation is consistent with the presentation and salvage of antigen presentation on cancer cells being a key factor for T cell recognition^37^.

We further applied PSAA to five SRT datasets of cancer TME. Using the PSAA predicted MHC-I level and expression level of CD8 + cytotoxic T cell markers, we identified spatial regions of high-/low-MHC-I level and high-/low-CTL cell level (See details in Methods). Strong consistencies between MHC-I presentation and CTL infiltration, evaluated by Moran’s I correlation, was identified in the TME of different cancer types (Supplementary Table S4). In addition, we identified a substantial number of distinct spatial regions of high MHC-I and low CTL infiltration (Fig. 4c and Supplementary Fig. 3b). However, we rarely observed spatial regions of low MHC-I and high CTL infiltration (Supplementary Fig. 3b). All the identified spatial spots of low MHC-I and high CTL infiltration are on the boundary of the high MHC-I and high CTL regions. Moreover, the numbers of spatial spots of high MHC-I and low CTL infiltration are consistently higher than the spots of low MHC-I and high CTL infiltration in all analyzed data (Supplementary Table S4). Our observations suggested that there were additional factors in these regions, such as stromal variations or metabolic shifts, inhibited the infiltration of CTLs. To identify the biological functions that are associated with low T cell infiltration, we utilized a generalized linear model to identify the genes that are consistently and specifically expressed in the regions of high MHC-I and low CTL versus the other regions in the analyzed data and downstream pathway enrichment analysis (see Methods).

We observed upregulated CTL-related pathways in high MHC-I and high CTL vs high MHC-I and low CTL regions, upregulated MHC-I genes in high MHC-I and low CTL vs low MHC-I and high CTL regions, and upregulated general adaptive immune responses in high MHC-I and high CTL vs low MHC-I and low CTL regions (Fig. 4d). We also identified new stromal, TME, and metabolic changes that may be related to low CTL-infiltration in high MHC-I regions. Upregulated ECM formation, coagulation cascades, myeloid cells including monocytes and granulocytes, and chemokine receptors were seen in high MHC-I and high CTL vs high MHC-I and low CTL regions. In addition, increased metabolic activities including glycolysis, TCA cycle, oxidative phosphorylation, electron transport chain (ETC), glycan and steroid synthesis were seen in (1) high MHC-I and low CTL vs low MHC-I and high CTL regions and (2) high MHC-I and high CTL vs low MHC-I and low CTL regions, suggesting possible roles of metabolic activity related to MHC-I presentation (Fig. 4d). We also observed down regulation of the TGBF-beta, JAK-Stat, glucose transport, glutathione metabolism, and histone deacetylase III pathways in high MHC-I and high CTL vs high MHC-I and low CTL regions.

To demonstrate the clinical implication of PSAA, we applied the method to the bulk RNA-seq data of a melanoma data set (GSE91061) collected from patients under anti-PD1 therapy using Nivolumab^38^. In total, we obtained 105 samples from this data set, including 48 partial response (PR), 34 stable disease (SD), and 23 progressive disease (PD) patients. We applied PSAA to compute sample-wise activity level of the eight steps in the MHC-I pathway. Biologically, we expect the higher level of antigen presentation activity is associated with better response. We adopted multi-variate logistic regression with a L1-penalty to identify the top variables and best model in predicting patients’ response to the ant-PD1 therapy. Considering that the quality of cancer associated antigen also determines CTL recognition, we included predicted microsatellite stability (MSS) or microsatellite instability (MSI) status as a confounding factor (see details in Supplementary Methods). In addition, we also introduced total T cell level and cytotoxic CD8 + T cell level predicted by deconvolution analysis and the on-/off-treatment status provided in the data as additional factors. The final selected model is:

$$Responsiveness\sim logistic\left(3.02•Peptide\;loading+1.12•MSI\;status-1.55\right)$$

, where *Responsiveness* is a binary variable that takes values in “responder” and “non-responder”, and the *p*-value of the peptide loading and MSI status are 6.4e-4 and 0.02, respectively, suggesting the activity of the peptide loading step has a higher power predicting the outcome of immuno-therapy than MSI status and T cell abundance.

We further checked how the predicted rate of peptide loading varies with respect to responsiveness, MSI status, and treatment status. We observed that the MHC-I antigen presentation level in the PR group is consistently higher than the SD and PD group, and the SD group is also higher than the PD group, in both MSS and MSI, and on-treatment/pre-treatment patient groups. Limited by sample size, a significant difference of the MHC-I antigen presentation level is only observed between the PR and SD groups vs the PD group in the on-treatment patient of MSS (Fig. 4e). Specifically, the MSS of PR and SD on treatment patients have a significant increase of predicted rate of peptide loading compared to (1) the MSS of PR patients on-treatment patients (p = 0.0026) and (2) the MSI of all PD and SD on-treatment patients (p = 0.022). Our observation suggests that PD-1 inhibitor has a better eficacy in the melanoma patients of low mutation load or low cancer-associated antigen quality if the cancer cells have a higher level of MHC-I presentation. Complete predicted activity level of MHC-I reactions and clinical information of each sample are given in Supplementary Table S5. To validate our observations, we also analyzed three additional datasets collected from melanoma (GSE115821^39^), lung cancer (GSE126043^40^), and cutaneous T cell lymphoma (GSE162137^41^) patients treated by PD-1 inhibitor. Increased MHC-I antigen presentation levels were detected in the responders compared to non-responding patients in all the datasets (Fig. 4f).

We further utilized the in-silico perturbation function of PSAA to identify the genes that could be targeted to improve the presentation level of MHC-I complex on the surface of a cell. Previous studies reported that *MAL2* in the salvage pathway of MHC-I is a critical negative regulator of MHC-I presentation ^37^. A leave-one-out statistical test evaluates the significance of change of the predicted flux balance when including or excluding *MAL2* in the recycling step of the MHC-I pathway (see details in Methods and Supplementary Table S6). Application of the test on TCGA data revealed that *MAL2* is significantly involved in the recycling step of the MHC-I pathway in seven cancer types.

To experimentally validate the PSAA predictions, we used in-silico perturbation analysis to predict genes that consistently negatively impact MHC-I antigen presentation using pan-cancer data from TCGA. Specifically, we added each gene into the recycling module to train the PSAA model and rank the genes according to their PSAA-predicted cell surface MHC-I level when perturbing their gene expression (Fig. 4g). To further validate the PSAA analysis, we knocked down the top predicted genes in mouse breast cancer cells and evaluated the antigen presentation level and T cell killing between knockdown vs control for the top 11 predicted genes. We observed that knocking down each of the predicted genes significantly increased MHC-I antigen presentation and T cell killing effect (range: 22–148%) (Fig. 4h). See experimental details in Methods.

### PSAA identified unaligned MHC-II antigen presentation steps in microglia and other cell types in Alzheimer’s disease brain.

Although the brain was initially considered as an immune-privileged site where antigen presentation would not occur, both microglia and astrocytes present antigens via MHC class II molecules to activate CD4 + T cells and stimulate immune responses in the central nervous system (CNS)^42–44^. However, the role of the immune system and the antigen presentation process is not well understood in neurodegenerative diseases, such as Alzheimer’s disease and Parkinson’s disease^45, 46^.

**Figure 5:**
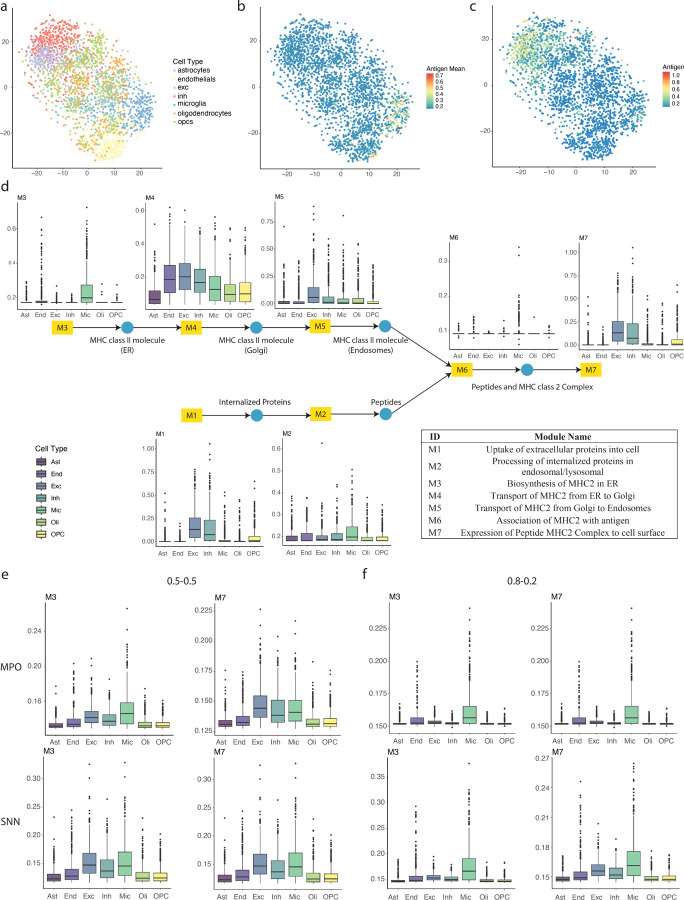
Application of PSAA on ROSMAP AD data. (a) tSNE plot of selected cells in ROSMAP scRNA-seq data. (b-c) Distribution of predicted activity level of (b) biosynthesis of MHC-II in ER and (c) expression of MHC-II complex on the surface of each cell. (d) Predicted activity level of each reaction step in each cell type using the default optimizer. (e-f) Predicted activity level of biosynthesis of MHC-II in ER and expression of MHC-II complex on the surface by the message passing step after and supervised learning step after burning-in using hyperparameters $Y_{M1}=\ldots =Y_{M7}=0.5$ (e) and $Y_{M3}=0.8,Y_{M1}=Y_{M2}=Y_{M4}=\cdots Y_{M7}=0.2$ (f).

To understand the cell type-specific MHC-II antigen presentation status in the AD brain, we applied PSAA on the ROSMAP AD scRNA-seq data to evaluate the MHC class II antigen presentation levels in different cell types. Cell type annotation was provided for the 172,659 cells, including seven cell types: microglia (Mic), astrocytes (Ast), endothelial cells (End), excitatory neurons (Exc), inhibitory neurons (Inh), oligodendrocytes (Oli), and oligodendrocyte precursor cells (OPC). For a better visualization, we randomly sample 500 cells of high total UMI from each cell type in the AD samples (Fig. 5a) and applied PSAA to estimate the MHC class II antigen presentation level in each cell (Fig. 5b, 5c). PSAA identified that microglia have the highest activities of the reactions involved in the ‘Processing of internalized proteins in endosomal/lysosomal’ and ‘Biosynthesis of MHC-II complex’ (Fig. 5d). However, microglia and astrocytes exhibit lower levels of further processing modules of MHC class II molecules compared to Exc and Inh (Fig. 5d). Compared to the antigen presentation cells in infectious disease and the TME of cancer, microglia in the AD brain have a much higher inconsistency between the MHC-II complex biosynthesis step and its processing and presentation onto cell surface. Thus, we hypothesize that even though microglia can express and produce MHC class II molecules, they still have a low rate of MHC-II complex presentation on the cell surface.

To further test this hypothesis, we adjusted the weight for the imbalance loss of each reaction step when applying the optimization algorithm, MPSL. Modulating these weights facilitates an extensive search for the potential solution space of the functions that could enhance the flux balance across the entire pathway. We observed that the iterations between the message passing optimization and supervised learning steps failed to yield a consistent distribution of the flux when the algorithm reached convergence (burns-in) across all tested hyperparameters (Fig. 5e, 5f). We also examined how the flux distribution responded to the changes of the hyperparameters. A higher level of MHC-II activity in microglia was predicted by the message passing step when using a higher weight of the flux balance loss for the “Biosynthesis of MHC-II complex” reaction (Fig. 5f). However, the flux predicted by the supervised learning step did not align with the message-passing-derived flux.

This inconsistency suggests that there is no function that could be identified by PSAA to map the ROSMAP AD brain scRNA-seq data onto the flux balance solution space of the MHC-II pathway, implying that brain cells do not adhere to the classic MHC-II pathway characteristic of immune cells. Our analysis indicates the potential existence of an alternative mechanism for peptide processing and loading in the MHC-II pathways, or a mismatch between peptide processing and MHC-II complex expression in the microglia in AD brains.

Systems biology characterizes the motion of molecules within a complex biological process as differential equation-based dynamic systems ^47, 48^. Recently, physics-informed neural networks (PINNs) have emerged as a powerful approach in this field, integrating neural network architectures with physical laws to model complex biological systems. A reliable systems biology model provides explicit quantification and interpretations of the system ^48, 49^, and enables simulation and perturbation analysis to study the impact of each biological feature and their interactive effects in the system ^48, 50, 51^. Despite a plethora of knowledge on the differential equation-based systems biology model, dynamic models are dificult to establish within specific biological contexts, especially when only static data is available. Although a challenge, the large amount of biological omics data has the potential to characterize complex biological systems.

Here we presented the PSAA framework that approximates the activity level and dynamics of MHC-I and MHC-II pathways by using multi-omics data. Compared to conventional systems biology and data-driven computational biology approaches, PSAA bridges omics data with explicit systems biology models by integrating neural network frameworks. Crucially, we demonstrate that for systems with steady-state equilibrium—such as metabolic pathways and macromolecule processing—dynamic behaviors can be inferred from non-time course omics data, expanding the applicability of PSAA to a broader range of biological data contexts. PSAA demonstrated that reaction rates within the system having equilibrium steady states can be approximated by properly designed neural networks, which forms a new type of physics informed neural network. We call this type of analysis PINN-empowered and data-driven systems biology and the underlying learning paradigm as constrained learning. Constrained learning is defined by (1) approximating the non-linear dependency between the dynamics of biological reactions and observed omics data by AI-based non-linear solvers such neural networks and (2) constraining the solution space (functional space) of the non-linear solver by the dynamic properties of the systems, i.e, a type of PINN. We provided a mathematical formulation of the general constrained learning problem (see details in Supplementary Methods).

PSAA illustrates a newly defined machine learning paradigm and PINN architecture, which falls outside of traditional supervised and unsupervised learning. We refer to this learning paradigm as constrained learning for data-driven systems biology. This approach is characterized by identifying the mathematical model to quantify the reaction rates within a given omics data set, while enforcing coherence between the mathematical property of the model and the systems biological property of the reactions being studied. Empowered by this idea, the PSAA framework provides the following unmet capabilities: (1) quantify sample-wise activity level of the whole process and individual biological steps of MHC-I and MHC-II pathways using bulk, single-cell, or spatially resolved transcriptomics or proteomics data; (2) compute the dependency of MHC-I antigen presentation with T cell infiltration and activity level, and other biological processes or clinical features; (3) dissection of spatial regions of varied T cell infiltration and antigen presentation levels in SRT data; (4) assess the impact of the expression change of each gene on the MHC-I and MHC-II pathways in each sample; (5) prediction of possible drugs to perturb the level of MHC-I and MHC-II antigen presentation; and (6) inference of the goodness of fit of the data to prior assumed biological pathway structure. Selected analyses including prediction accuracy and targets predicted by in-silico perturbations have been validated on our in-house generated and public domain data sets.

Figure 3h, 4a and Supplementary Fig. 3a suggested that different samples may have varied levels of T cell activation. It is noteworthy that PSAA only models the flux rate of MHC-I/II pathway in a cell or tissue sample. T cell activation depends on the interaction of a specific TCR with the peptide-MHC complex and engagement with other co-stimulatory molecules. However, the TCR activation reaction, which is a signaling procedure, does not satisfy the condition of steady-state equilibrium. Thus, PSAA cannot effectively handle variations in TCR differences. In each training of the PSAA model, it is assumed that all samples share the same rate of T cell recognition and activation. One future direction is to extend the current PSAA by including parameters of TCR recognition and T cell activation to better characterize the interactions between APCs and T cells.

## Methods

### Public Data used in the analysis.

Reaction information of the genes involved in each step of MHC-I and II pathways were collected from KEGG, Reactome, GO databases and literature data. Detailed information of the pathway reconstruction was given in Supplementary Methods.

We collected three CITE-seq data sets, GSE249542, GSE200417^29^, and SCP1064^28^, from the GEO database to validate the predication accuracy of PSAA. Bulk RNA-seq and proteomics data of cancer cell lines from CCLE and cancer tissue data from TCGA were utilized to validate the PSAA predictions. RPKM normalized RNA-seq data and normalized proteomics data were downloaded from cBioPortal. Cancer types were selected based on the availability of normal samples in TCGA. We retrieved five spatial transcriptomics slides of cancer tissue from the 10x Genomics website and GSE206522 to validate the application of PSAA to cancer data. Four transcriptomics data of patient samples collected from clinical trials of anti-PD1/CTLA-4 therapies, GSE91061^38^, GSE115821^39^, GSE126043^40^, and GSE162137^41^, were collected from the GEO database to demonstrate the clinical implications of PSAA predicted antigen presentation levels. ROSMAP scRNA-seq data of AD brain was retrieved from ROSMAP data portal. Detailed information of data retrieval, processing, and normalization were given in Supplementary Methods.

Detailed experimental procedures of the scRNA-seq data of *H. ducreyi* infection from four human volunteers and matched spatial transcriptomics data, including sample collection, processing, library construction, and sequencing, were given in Supplementary Methods. The data was deposited to dbGaP (phs003754).

#### Mathematical formulation of PSAA

PSAA utilizes a directed factor graph base representation of the MHC-I or II pathway and a PINN model for reaction rate estimation. We first reconstruct the MHC-I or II pathway into a directed factor graph, in which each reaction $\left(R\right)$ is a variable, and each intermediate molecule such as peptides and MHC complex $\left(C\right)$ are factors.

Denote $FG\left(C,R,E_{C\to R},E_{R\to C}\right)$ as the factor graph, where C is the set of intermediate molecules, R is the set of reactions, $E_{C\to R}$ and $E_{R\to C}$ are direct edges represent R consumes or produces C. Denote ${𝓡}_{j}^{m}=\left\{R_{m}|\left(R_{m}\to C_{k}\right)\in E_{C\to R}\right\}$ and ${𝓡}_{out}^{C_{k}}=\left\{R_{m}|\left(C_{k}\to R_{m}\right)\in E_{R\to C}\right\}$ as the sets of the reactions $C_{k}$ or consume $C_{k}$. For an omics data of N samples, denote $X_{j}^{m}=\left\{x_{1,j}^{m},\ldots ,x_{i_{m},j}^{m}\right\}$ as the expression of the genes or proteins involved in the reaction $R_{m}$ and $F_{m,j}$ as the rate of $R_{m}$ in sample j. We model $F_{m,j}=𝓕\left(X_{j}^{m},\Theta_{m}\right)$ as a multi-layer neural network with the input $X_{j}^{m}$, $\Theta_{m}$ denotes the parameter of the neural network. $\Theta_{m}$ and cell-wise flux $F_{m,j}$ are solved solvedby minimizing the loss:

$$\sum_{j=1}^{N}\sum_{k=1}^{K}\gamma_{k}{\left(\sum_{m\in {𝓡}_{in}^{C_{k}}}F_{m,j}-\sum_{m^{\prime }\in {𝓡}_{out}^{C_{k}}}F_{m^{\prime },j}\right)}^{2}+L_{p}\left(𝓕,\Theta \prime \right)$$

Here the first loss term regularizes the coherency to the flux balance condition of the biological system on the observed data. A quadratic loss was used to enable certain imbalances for the samples under quasi-steady state or non-steady state. Parameter $\gamma_{k}$ is introduced in PSAA to enable different weights for the flux balance of different steps. Noted, the whole network of antigen processing, presentation, and recognition is composed by three main branches, namely (1) peptide generation (ubiquitination and proteosome), (2) peptide loading, and (3) processing and exocytosis of MHC class I or II complexes, where only the step (2) is antigen presentation specific while the activity level of (1) and (3) could be regulated for other biological activities that the presentation of MHC complexes. Thus, $\gamma_{k}$ is introduced to leverage the influence of varied expression levels of different steps in antigen presentation. We demonstrated the proper value range of $\gamma_{k}$ can be identified by maximizing the best fitting of the data to the underlying systems biology model of the antigen presentation pathways. Detailed systems biology consideration is provided in Supplementary Methods.

#### MPSL optimization algorithm

The solution of ▯ is non-trivial because it needs to search over the functional space of ${𝓕}_{m}\left(X_{j}^{m},\Theta^{m}\right)$ and parameter space of $\Theta^{m}$ for each reaction m. In PSAA, we utilize neural networks to approximate the non-linear dependency between omics data and reaction rate ^27^. Noted, minimization of the loss term ▯ neither falls into the paradigm of supervised learning nor unsupervised learning. Instead, the loss term characterizes the biochemical dependencies among 𝓕. Furthermore, the sparse learning penalty term $L_{p}\left(𝓕,\Theta^{\prime }\right)$ cannot be directly co-optimized with the flux balance term by using existing methods. Thus, we developed a new optimization algorithm for ▯, namely Message Passing and Supervised Learning (MPSL). Specifically, considering the flux balance term as additional constraints of the solution space of ${𝓕}_{m}$, we proposed an iterative approach to effectively search for the solution space. Because the flux balance constraint could be represented as factors over an directed factor graph, in which each function 𝓕 is a variable and Information balance over a factor can be achieved by belief propagation ^52^. Based on this idea, we develop a three-step optimizer to minimize ▯:

1. **Step 1 (Initialization):** Classic gradient decent is first utilized to generate an initial solution $𝓕\left(D_{0},\Theta_{0}^{\prime }\right)$
2. **Step 2 (Message Passing Optimization, MPO):** for any given $𝓕\left(D_{0},\Theta_{0}^{\prime }\right)$ with fixed $D_{0}$ and $\Theta_{0}^{\prime }$ the optimization strategy adopts the idea of belief propagation (BP) to identify a solution ${𝓕}^{\prime }$ which is on the solution space of $\Phi \left(𝓡\left(\Theta \right)\right)$ and close to $𝓕\left(D_{0},\Theta_{0}^{\prime }\right)$. Noted, here BP only searches ${𝓕}^{\prime }$ only based on the flux rates $𝓕\left(D_{0},\Theta_{0}\right)$ and does not rely on $D_{0}$. ${𝓕}^{\prime }$ can be considered as projecting the values of $𝓕\left(D_{0},\Theta_{0}\right)$ to the solution space that minimizes the flux balance term.
3. **Step 3 (Supervised Learning):** Then ${𝓕}^{\prime }$ could serve as known labels of flux rate of each reaction. 𝓕 and $\Theta^{\prime }$ could be further updated by a supervised fitting of $𝓕\left(D_{0},\Theta^{\prime }\right)$ to ${𝓕}^{\prime }$ for all data points $D_{0}$. Under default setting, $𝓕\left(D_{0},\Theta^{\prime }\right)$ will be trained as a fully connected deep neural network to predict ${𝓕}^{\prime }$ and $L_{1}$ penalty will be used for $L\left(𝓕,\Theta^{\prime }\right)$.

Step 2 and 3 could be iteratively conducted to iteratively optimize ${𝓕}^{\prime }$ and $\left\{𝓕,\Theta^{\prime }\right\}$. It is noteworthy that BP can effectively handle linear constraints such as flux balance under quasi-steady state. By using this approach, the two terms could be iteratively and effectively handled, and the $L_{p}\left(𝓕,\Theta^{\prime }\right)$ can be directly minimized in the supervised learning form in Step 3.

#### In-silico perturbation analysis to evaluate the impact of each gene

To study which gene contributes most to MHC class II antigen presentation pathways in APCs, we conduct an in-silico perturbation analysis. The first order partial derivative of each gene in the PSAA model was computed for each sample. We first got the absolute value of the first order partial derivative and then used log transformation to make sure that all the gradients are positive values. After that, we performed principal component analysis (PCA) in a gradient matrix and selected the top 6 PCs to represent the importance of each gene. Then we used k-means clustering to cluster the importance matrix into three clusters as elaborated in the main text. Finally, we ranked genes based on their importance within each cluster and identified the top 10 genes in each cluster for further analysis.

#### Identification of gene targets to improve the antigen presentation level

To identify the genes that could be targeted to improve the presentation level of MHC-I complex on the surface of a cell, we evaluate if adding a certain gene into the “MHC-I complex salvage” module could significantly increase the balance of the fitting. Specifically, we performed a paired Wald test using TCGA data of TNBC and other cancer types. We define the flux imbalance loss with target gene as $L_{X}=\left(l_{1}^{X},l_{2}^{X}\ldots l_{n}^{X}\right)$ and the flux imbalance loss without target gene as $L_{Y}=\left(l_{1}^{Y},l_{2}^{Y}\ldots l_{n}^{Y}\right)$. The null hypothesis test is $H_{0}:\mu_{X}-\mu_{Y}=0$. The alternative hypothesis is $H_{a}:\mu_{X}-\mu_{Y}<0$. We tested a list of target genes and reported their p-values in Supplementary Table S6. The CRISPR knock experiment of the genes with most significant p-values were conducted on a TNBC cell line system as detailed below.

#### Cell culture and Generation of stable knockdown cell lines of the CRISPR knockdown experiment

The mouse breast cancer cell line with endogenous expression of ovalbumin, EO771-OVA, was maintained in DMEM medium supplemented with 10% fetal bovine serum and 1% penicillin/streptomycin at 37°C with 5% CO2. For the generation of stable shRNA knockdown cell lines, shRNA clone sets targeting different mouse genes were purchased from Sigma and transduced into EO771-OVA cells via lentivirus, followed by 2 µg/ml puromycin selection for 5 days. qPCR was used to examine the knockdown efficacy.

#### Antigen presentation and cytotoxicity assay to validate thepredicted genes

Antigen presentation levels on EO771-OVA and knockdown cell lines were examined using APC-conjugated anti-mouse H-2Kb bound to SIINFEKL antibody (BioLegend, Dilution 1:50) and evaluated by flow cytometry. EO771-WT cells were used as isotype control for gating strategy.

To assess the CD8 + T cell cytotoxicity against EO771-OVA cell lines with gene knockdown, CD8 + T cells were isolated from the splenocytes of OT-I mice and stimulated with CD3/CD28 dynabeads (Gibco^™^ #11131D) in the presence of 5 ng/mL IL-2 for 2 days. EO771-OVA cells were stained with IncuCyte Cytolight Rapid Red (Sartorius, #4705) and seeded in 96-well plate (5 × 10^3^ cell/well). OT-1 T cells were co-cultured with EO771-OVA cells at effector/tumor-cell ratio of 3:1. The 16h time point was used for final readout.

##### Signal amplification of spatial transcriptomics data.

To reduce the discontinuities inherent caused by the low signal level, we employed a Gaussian smoothing filter to amplify the spatial dependent signals in SRT data, such as the predicted activity level of antigen presentations or T cell abundance. Specifically, the following smoothing model was used to amplify spatial dependent signals:

$$f_{i}^{amp}=\sum_{j\in N\left(i\right)}g\left(d\left(i,j\right)\right)*f_{i}$$

, where $g\left(x\right)=\frac{1}{\sigma \sqrt{2\pi }}e^{-\frac{{\left(x-\mu \right)}^{2}}{2\sigma^{2}}}$ is a gaussian kernel and $f_{i}$ represents the PSAA predicted antigen presentation level or original T cell expression level of spot i. $N\left(i\right)$ is the neighborhood of i defined as the spatial spots whose distance to i are smaller than a certain threshold, and $d\left(i,j\right)$ is the Euclidean distance between two spatial spots i and j, and $f_{i}^{amp}$ is the amplified signal of spot i.

##### Identify spatial regions of varied dependency between antigen presentation and T cell infiltration.

To evaluate the spatial dependency between antigen presentation and T cell infiltrations and identify the spatial regions show varied dependencies, we computed local bivariate Moran’s I correlation^53^ for each spatial spot i:

$$I_{i}=\frac{\sum_{j}w_{ij}y_{j}\times x_{i}}{\sum_{i}x_{i}^{2}}$$

where $x_{i}$ represents the normalized antigen presentation level after amplification at spot i, $y_{j}$ represents the normalized T cell level after amplification at spot j, and $w_{ij}$ is a weight indexing location of spot i relative to j. In this study, we used the gaussian kernel distance to compute $w_{ij}$.

For each spot i, $I_{i}$ measures the correlation between its antigen presentation level $\left(x_{i}\right)$ with the level of T cell abundance $\left(y_{i}\right)$ in its neighborhood $\left(\sum_{j\in N\left(i\right)}w_{ij}y_{j}\right)$. The significance of $I_{i}$ was evaluated using a permutation test, where the y values were randomly permuted across all spots. A pseudo p-value was then calculated by determining the proportion of Local Moran’s I values generated from permutations that are greater than or equal to the observed Local Moran’s I values from original data. We set significant level α=0.05 and the spots with pseudo p-value p<0.05 were considered statistically significant, indicating significant a spatial dependence between antigen presentation level and T cell infiltration level.

Then we segment the regions of significant dependencies into four regions based on the level of antigen presentation and T cell signals – “High antigen, High T cell”, “High antigen, Low T cell”, “Low antigen, High T cell” and “Low antigen, Low T cell”. To conduct this segmentation, we first normalize the antigen presentation level of each spot by computing the z-score of neighbors by the weighted average z-score of $x_{i}$, denoted as $z\left(x_{i}\right)$, and the T cell infiltration level of its neighbors by the weighted average z-score of $y_{j}$, denoted as $w_{ij}z\left(y_{j}\right)$. The “High” or “Low” antigen and “High” or “Low” T cell regions were identified by positive or negative $z\left(x_{i}\right)$ and positive or negative $w_{ij}z\left(y_{j}\right)$), respectively.

## Supplementary Material

Supplement 1
