# Supplementary material for: A physics informed neural network approach to quantify antigen presentation activities at single cell level using omics data
Source: Res Sq. 2025 Jan 17:rs.3.rs-5629379. Preprint. [Version 1] doi: 10.21203/rs.3.rs-5629379/v1 (PMC11774464; doi:10.21203/rs.3.rs-5629379/v1)
Supplement: Supplement 1 [file NIHPPRS5629379v1-supplement-1.pdf]

Supplementary Files

This is a list of supplementary files associated with this preprint. Click to download.

- Supplementarymethodssubmission.docx
- SupplementaryTableS1.xlsx
- SupplementaryTableS2.xlsx
- SupplementaryTableS3.xlsx
- SupplementaryTableS4.xlsx
- SupplementaryTableS5.csv
- SupplementaryTableS6.xlsx
- SupplementaryFigure1.pdf
- SupplementaryFigure2.pdf
- SupplementaryFigure3.pdf
